# Tagitinin C induces ferroptosis through PERK-Nrf2-HO-1 signaling pathway in colorectal cancer cells

**DOI:** 10.7150/ijbs.59404

**Published:** 2021-06-26

**Authors:** Ruiran Wei, Yueqin Zhao, Juan Wang, Xu Yang, Shunlin Li, Yinyuan Wang, Xingzhi Yang, Jimin Fei, Xiaojiang Hao, Yuhan Zhao, Liming Gui, Xiao Ding

**Affiliations:** 1 Center for Tissue Engineering and Stem Cell Research, Guizhou Medical University, 550004, Guiyang, China.; 2 State Key Laboratory of Phytochemistry and Plant Resource in West China, Kunming Institute of Botany, Chinese Academy of Sciences, 650201, Kunming, China.; 3 Yunnan Cancer Hospital & The Third Affiliated Hospital of Kunming Medical University, 650118, Kunming, China.

**Keywords:** ferroptosis, tagitinin C, ROS, ER stress, Nrf2-HO-1 pathway

## Abstract

**Rationale:** Colorectal cancer (CRC) is a common malignant tumor of the digestive system. However, the efficacy of surgery and chemotherapy is limited. Ferroptosis is an iron- and reactive oxygen species (ROS)-dependent form of regulated cell death (RCD) and plays a vital role in tumor suppression. Ferroptosis inducing agents have been studied extensively as a novel promising way to fight against therapy resistant cancers. The aim of this study is to investigate the mechanism of action of tagitinin C (TC), a natural product, as a novel ferroptosis inducer in tumor suppression.

**Methods:** The response of CRC cells to tagitinin C was assessed by cell viability assay, clonogenic assay, transwell migration assay, cell cycle assay and apoptosis assay. Molecular approaches including Western blot, RNA sequencing, quantitative real-time PCR and immunofluorescence were employed as well.

**Results:** Tagitinin C, a sesquiterpene lactone isolated from *Tithonia diversifolia*, inhibits the growth of colorectal cancer cells including HCT116 cells, and induced an oxidative cellular microenvironment resulting in ferroptosis of HCT116 cells. Tagitinin C-induced ferroptosis was accompanied with the attenuation of glutathione (GSH) levels and increased in lipid peroxidation. Mechanistically, tagitinin C induced endoplasmic reticulum (ER) stress and oxidative stress, thus activating nuclear translocation of nuclear factor erythroid 2-related factor 2 (Nrf2). As a downstream gene (effector) of Nrf2, heme oxygenase-1 (HO-1) expression increased significantly with the treatment of tagitinin C. Upregulated HO-1 led to the increase in the labile iron pool, which promoted lipid peroxidation, meanwhile tagitinin C showed synergistic anti-tumor effect together with erastin.

**Conclusion:** In summary, we provided the evidence that tagitinin C induces ferroptosis in colorectal cancer cells and has synergistic effect together with erastin. Mechanistically, tagitinin C induces ferroptosis through ER stress-mediated activation of PERK-Nrf2-HO-1 signaling pathway. Tagitinin C, identified as a novel ferroptosis inducer, may be effective chemosensitizer that can expand the efficacy and range of chemotherapeutic agents.

## Introduction

Colorectal cancer is the fourth most common cancer cause of death globally, with about 2, 000, 000 new cases and 600, 000 deaths per year. The recorded incidence of colorectal cancer has been on the increase in developed countries 1. Unlike other cancers, such as lung cancer, there is no single risk factor for colorectal cancer. Epidemiological studies have identified that many factors have contributed to this increase, such as age and sex, inflammatory bowel disease, lifestyle or environmental changes 2. A total mesorectal excision is standard surgical procedure for the treatment of colorectal cancer, however, that radical surgery and chemotherapy lead to severe side effects, such as reduced immune function and reduction in quality of life. And immunotherapy has been applied to a subset of colorectal cancers, with median progression-free survival (PFS) rate of 61% and overall survival (OS) rate of 66% at 24 months 3. So far, over half of all sufferers do not survive for longer than 5 years after diagnosis, despite it being curable if diagnosed early. In the clinic, to integrate novel targeted therapeutic agents into standard therapy is key for increasing the survival of patients and improving overall quality of life. This necessitates further insight into colorectal cancer and discovery of new drug(s) 4.

Ferroptosis is an iron- and ROS-dependent programmed cell death that is distinct from apoptosis, autophagy, and necrosis at morphological, biochemical, and genetic levels 5,6. Emerging studies have discovered that ferroptosis is a new therapeutic strategy to overcome the drug resistance in cancer cells 7,8. Whether as monotherapy or in combination with other chemotherapeutic drugs, ferroptosis inducers can induce ferroptosis in cancer cells, especially in drug-resistant cancer cells 9. In addition, ferroptosis can selectively target aggressive cancer stem cells and is also expected to enhance the efficacy of immunotherapy and overcome the resistance to immunotherapy 10,11. Erastin is a ferroptosis inducer that functionally inhibits cystine-glutamate reverse transporter (System Xc-). Cystine uptake would be inhibited by erastin, which leads to deprivation of the antioxidant glutathione and eventually oxidative cell death 12,13.

Ample studies have suggested that some natural plant extracts show significant antitumor effect through ferroptosis, including artemisinim, amentoflavone, ruscogenin, gallic acid, dihydroisotanshinone I and so on 14-19. Tagitinin C is a sesquiterpene lactone which is widely found in Asteraceae 20. It has increasingly attracted the interests of researchers because of its various pharmacological activities including antitumor, antiviral, antifibrotic, antiparasitic and cardioprotective effects 21-23. It has been reported that tagitinin C induces the cell death through triggering autophagy in human glioblastoma cells 24; and in hepatocellular carcinoma (Hep-G2), tagitinin C arrests the cell cycle at sub-G1 and S phase and induces apoptosis 25.

Herein, we found that tagitinin C has the ability to activate ferroptosis pathway in the colorectal cancer cells. Previous reports have pointed out some tumor cell lines were insensitive to ferroptotic inducers 26. In line with this, our experiments showed that colorectal cancer line HCT116 cells were insensitive to erastin‐induced ferroptosis. We therefore used these cells to explore the feasibility that tagitinin C could induce and/or enhance the incidence of ferroptosis and elucidate its mechanism. Our results demonstrated that cell viability of HCT116 cells was significantly reduced by tagitinin C. Evidence of ferroptosis features in tagitinin C treated cells, including a high production of ROS and lipid peroxidation. Mechanistically, tagitinin C induced ferroptosis through ER stress-mediated PERK-Nrf2-HO-1 signaling pathway. The combination of tagitinin C and erastin synergistically activated ER stress and enhanced ferroptosis. Our current study sheds light on development of a potential treatment strategy for CRC, especially chemoresistant CRC, with combinational therapy including tagitinin C.

## Materials and Methods

### Cell culture

CRC cell lines were cultured in DMEM or RPMI‐1640 (Gibco, Thermo Fisher Scientific, Germany) supplemented with 10% fetal bovine serum (Gibco, Thermo Fisher Scientific, Germany), 1% antibiotics (penicillin 10000 U/ml, streptomycin 100 mg/ml) (Solarbio, Beijing, China). The cells were maintained at 37 °C in a humidified atmosphere of 5% CO_2_, and were used during their logarithmic growth phase.

### Cell viability assay

The cytotoxicity caused by tagitinin C was measured by the 3-(4,5-dimethylthiazol-2-yl)‐2, 5-diphenyltetrazolium bromide (MTT) assay (Meilunbio, Dalian, China) or CCK-8 assay (APE × BIO, USA). Cells were seeded in 96-well plates in 100 μL medium. 20 μL MTT or 10 μL CCK-8 solution were added to each well and incubated at 37 °C for 4 h. The absorbance at 490 or 450 nm were measured on a spectrophotometer.

### Clonogenic assay

500 cells/well were seeded into a 6-well plate and treated for 14 days with tagitinin C. Then th**e** clones were stained with 1% crystal violet solution (Solarbio, Beijing, China) for 30 min. After washing with PBS for three times, the colonies were imaged by the camera and quantified. For estimation of the number of colonies formed, crystal violet was dissolved in 100% methanol, transferred to a 96-well plate, diluted 1:10 using PBS and absorbance was measured at 540 nm using spectrophotometer.

### Transwell migration assay

Cell migration was examined by a transwell chamber apparatus (24-well plates, 8-μm pore size. Corning Incorporated Costar, USA). Briefly, the lower chamber was filled with 750 µl RPMI-1640 containing 10% FBS. A total of 2×10^4^ cells in 200 µL serum-free RPMI-1640 were seeded in the upper well and were respectively incubated with tagitinin C for 24 h at 37 °C. Migrated cells were fixed with 4% paraformaldehyde and stained with 1% crystal violet. Images were captured using a microscope and the migrated cells were counted.

### Annexin V-FITC-propium iodide assay

An Annexin V-FITC apoptosis kit (Meilunbio, Dalian, China) was used to determine the number of apoptotic cells according to the manufacturer's instructions. Briefly, cells were grown in 6-well plates. When the cells reached 80-90% confluence, different treatments were applied to the cells. The cells were then harvested, washed twice with ice-cold PBS and resuspended in 400 μL of binding buffer. Then, 5 μL Annexin V and 5 μL propidium iodide were added and the mixture was incubated in the dark at 37 °C for 30 min. A total of 10,000 events per sample were analyzed. Annexin V positivity was calculated with a FACS Calibur flow cytometer using Flowjo V10 software.

### Cell cycle assay

Cell Cycle Analysis Kit (Meilunbio, Dalian, China) was used to analyze cell cycle after treating with different concentration of tagitinin C (5, 10, 20 µM) according to the manufacturer's protocol. Briefly, cells were fixed by 70% ethanol at -20 °C for overnight, and washed with PBS for three times and stained with staining solution of the kit supplemented with 200 mg/ml RNase at 37 °C for 30 min in the dark. Then cell cycle rates were measured by flow cytometry and the percentage of cells at G0/G1, S, or G2/M phase were quantified.

### Measurement of lipid peroxidation

The total cellular lipid peroxidation was measured using a C11 BODIPY (581/591) probe (Cayman Chemical, USA). Cells were treated as indicated and when then incubated for 1 h at 37 °C with C11 BODIPY (2 µM) in fresh medium. Excess C11 BODIPY was removed by washing the cells twice with PBS. Labeled cells were then trypsinized and resuspended in PBS for flow cytometry analysis. Oxidation of the polyunsaturated butadienyl portion of C11 BODIPY resulted in a shift of the fluorescence emission peak from ~590 nm to ~510 nm proportional to lipid peroxidation generation and was analyzed using a flow cytometer (BD Biosciences, US).

### Measurement of malondialdehyde (MDA)

Cell malondialdehyde (MDA) assay kit (Beyotime, Shanghai, China) was used to measure cellular MDA contents based on thiobarbituric acid (TBA) reactivity. Briefly, HCT116 cells were seeded into 6-well plates and cultured overnight. After treated as indicated, cells were harvested and lysed. Then, after protein quantification of the lysate, MDA working solution was added and heated at 100 °C for 15 min. Next, the supernatant was collected after centrifuging at 1000 rpm for 10 min at 4 °C, and then measured at 532 nm with a microplate reader. The relative cellular MDA concentration was presented as percentage of control.

### Determination of the labile iron pool

The total cellular labile iron pool was detected based on the calcein-acetoxymethyI ester (C-AM) method. After trypsinization, the cells were washed twice with PBS followed by incubation of 2 µM calcein-acetoxymethyI ester (GLPBIO, USA) for 30 min at 37 °C. Then, the cells were washed with PBS and incubated with or without deferoxamine (5 µM) for 1 h at 37 °C. The cells were analyzed by flow cytometry or SpecttaMax iD3. Calcein was excited at 488 nm, and fluorescence was measured at 525 nm. The levels of the labile iron poor were calculated by the difference in cellular mean fluorescence with and without deferoxamine incubation.

### Measurement of ROS

DCFH-DA (Meilunbio, Dalian, China) was used to detect the ROS levels according to the manufacturer's protocol. In brief, cells were seeded in 6-well plates and cultured with tagitinin C. After treatment, cells were harvested and washed twice with PBS and labeled with 20 µM DCFH-DA under 37 °C for 30 min in the dark, cells were then collected and the fluorescence intensity of DCF was tested by SpectraMax iD3 or flow cytometry (BD Biosciences, US).

### Measurement of glutathione

Glutathione was measured using monochlorobimane (MCB) (Sigma-Aldrich, USA). The cells were plated in a black 96-well plate and treated with tagitinin C. Then cells were incubated for 30 minutes at 37 °C with MCB (32 µM) in PBS. Fluorescence was measured using SpecttaMax iD3 with excitation set at 390 nm and emission set at 478 nm.

### Mitochondrial membrane potential (ΔψM) assay

Mitochondrial membrane potential (ΔΨm) was measured using TMRE (MCE, Shanghai, China). In brief, when the ΔΨm is high, TMRE gathers in the mitochondria and produces red-orange fluorescence. HCT116 cells were seeded in 96‐well plates and treated with tagitinin C. Then cells were incubated for 30 minutes at 37 °C with TMRE (1 µM) in PBS. Fluorescence intensity was measured by microplate reader (Bio-Rad, Shanghai, China). The excitation wavelength was 540 nm, and the emission wavelength was 595 nm.

### Western blot analysis

Cells were collected and then lysed on ice for 30 min using radioimmunoprecipitation assay (RIPA) buffer (Meilunbio, Dalian, China). Equal amounts of cell lysates were subjected to electrophoresis in SDS-PAGE gel and transferred into PVDF transfer membranes for antibody blotting. The membranes were blocked with 5% nonfat dry milk for 1 h at room temperature (RT) and incubated with antibodies against ER stress-related proteins (Cell Signaling Technology, Germany), Nrf2 and HO-1 (Abcam, USA) overnight at 4 °C, and then incubated with horseradish peroxidase‐conjugated secondary antibody for 1 h. The ECL reagent was used for detecting target bands.

### Quantitative real‐time polymerase chain reaction (qPCR)

Total RNA was extracted from cells by TRIzol. Reverse transcription was carried out according to the manufactures' direction. Real-time PCR was carried out using SYBR GreenER qPCR superMix Universal and the A100 PCR system. Sequences of real-time PCR (qPCR) primers: Nrf2: 5′‐CACATCCAGTCAGAAACCAGTGG‐3′ and 5′‐GGAATGTCTGCGCCAAAAGCTG‐3′; HO-1: 5′‐5′‐AAGACTGCGTTCCTGCTCAAC‐3′ and 5′‐AAAGCCCTACAGCAACTGTCG‐3′; POR: 5′‐GGTGGCCGAAGAAGTATCTCT‐3′ and 5′‐AACCAGTAGGTTAGGAGACCC‐3′; GPX4: 5′‐GAGGCAAGACCGAAGTAAACTAC‐3′ and 5′‐CCGAACTGGTTACACGGGAA‐3′; FSP1: 5′‐AGACAGGGTTCGCCAAAAAGA‐3′ and 5′‐CAGGTCTATCCCCACTACTAGC‐3′; Actin: 5′-GATCTGGCACCACACCTTCT-3′ and 5′-GGGGTGTTGAAGGTCTCAAA-3′.

### Immunofluorescence

HCT116 cells were plated on coverslips and treated with 20 µM tagitinin C. Immunofluorescence was performed as the recommended protocol. Briefly, after washing twice in PBS, cells were fixed in methanol for 5 min at RT. After fixation, cells were washed three times in PBS for 5 min and treated with a blocking solution (1% BSA in PBS) for 30 min. Subsequently, the cells were washed twice in PBS and incubated with the primary antibody directed against Nrf2 (anti-rabbit, Abcam, USA) overnight at 4 °C. Then, cells were washed three times in PBS for 5 min and incubated for 2 h with a conjugated secondary antibody, goat anti-rabbit IgG-Cy3 (Thermo Fisher Scientific, USA). Nuclei were stained with DAPI Stain Solution. The images were captured using a Leica Confocal Microscope TCS SP8 (Leica Microsystems).

### Statistical analysis

All experiments were conducted at least three times, and the data are presented as the mean ± SD. Statistical significance among different treatments were determined by student's *t*-test. A *p* value < 0.05 was considered to be statistically significant.

## Results

### Tagitinin C suppresses cell growth and induces cell death

**Figure 1:**
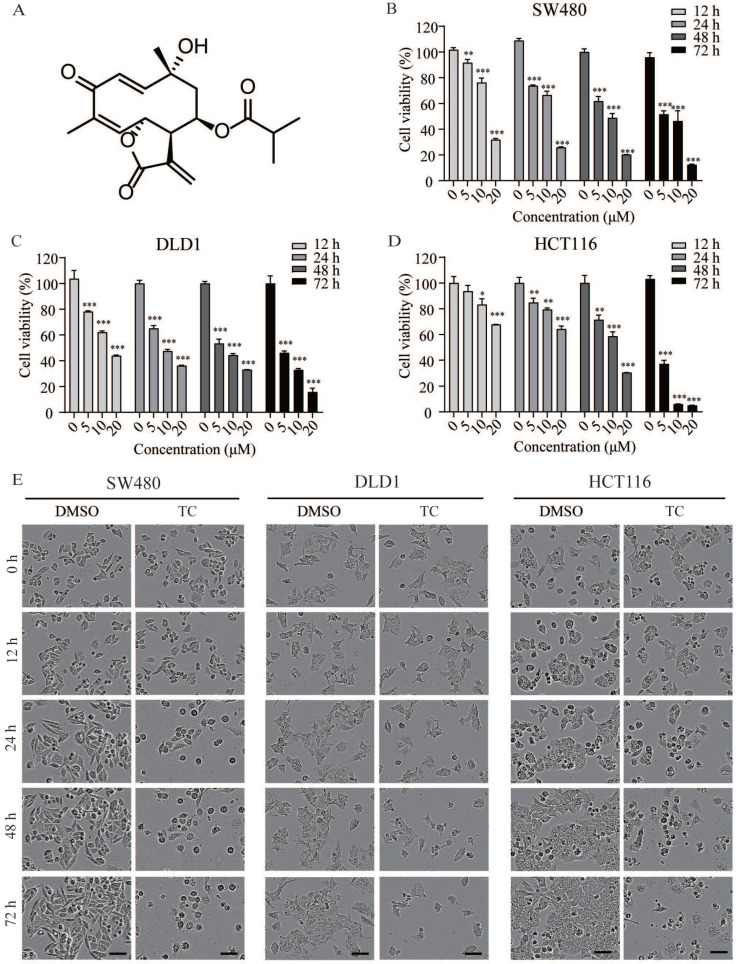
** Tagitinin C suppresses cell growth and induces cell death. (A)** The chemical structure of tagitinin C. **(B-D)** Cell viability of SW480, DLD1, and HCT116 cells were measured by MTT assay after treatment with indicate concentration of tagitinin C (5, 10, 20 µM) at 12, 24, 48, 72 h. **(E)** Cell morphology of SW480, DLD1, and HCT116 after treatment with concentration of tagitinin C (20 µM) at 0, 12, 24, 48, and 72 h (magnification, ×10). Data were presented as Mean ± SD. Scale bar indicates 40 µm. Statistical analysis was carried out between tagitinin C-treated group and DMSO group: * *p* ≤ 0.05, **p ≤ 0.01, *** p ≤ 0.001.

The chemical structure of tagitinin C is shown in Figure 1A. To determine the cytotoxic effect of tagitinin C on the proliferation of CRC cell lines, we treated colorectal cancer SW480 (Figure 1B), DLD1 (Figure 1C), and HCT116 (Figure 1D) cell lines with different concentrations of tagitinin C for 12, 24, 48, 72 h and assessed cell viability via MTT assay. The results showed that tagitinin C significantly reduced the viability of these three types of CRC cell lines in a concentration- and time-dependent manner compared to the untreated cells. Under microscopy, morphology of SW480 cells, DLD1 cells, and HCT116 cells were shattered, amorphous and multidirectional after tagitinin C treatment for different time points, which showed that tagitinin C treatment led to cell death of SW480, DLD1 and HCT116 cells (Figure 1E). In HCT116 cells, higher cell confluence conferred resistance to ferroptosis 27, however, we found that the HCT116 cell line is insensitive to erastin-induced ferroptosis (Figure S1A). Thus, we chose HCT116 cells for further study.

### Tagitinin C inhibits colony formation, cell migration abilities and induces G2/M phase cell cycle arrest in HCT116 cells

**Figure 2:**
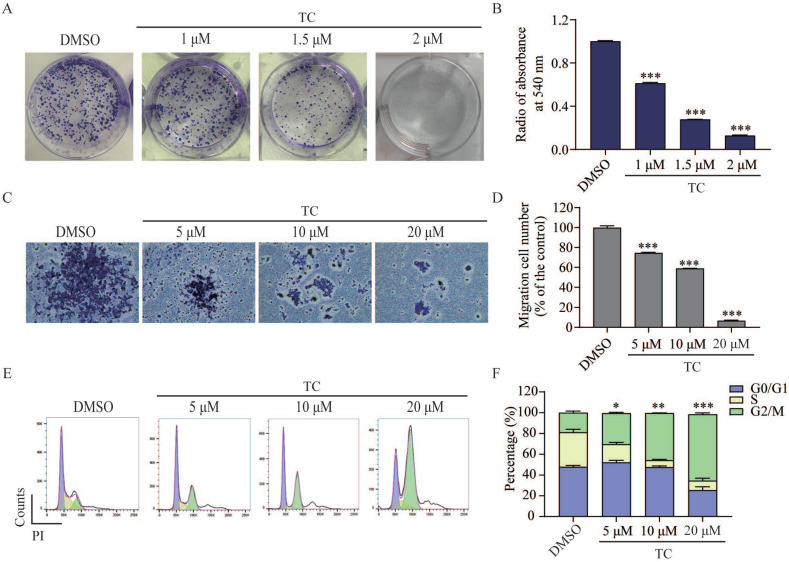
** Tagitinin C inhibits the colony formation, cell migration abilities and induces G2/M cell cycle arrest in HCT116 cells. (A-B)** Tagitinin C inhibited the colony formation ability of HCT116 cells in a dose-dependent manner. **(A)** Representative images of cell colonies after 14 days treatment tagitinin C (1, 1.5, 2 µM). **(B)** Bar graph shows the quantification of colonies numbers by measuring absorbance at 540 nm. **(C-D)** Tagitinin C inhibited the migration ability of HCT116 cells. **(C)** Transwell migration assay was performed in HCT116 cells treated with tagitinin C (5, 10, 20 µM) and representative images were shown. **(D)** Quantitative analysis for number of migrated HCT116 cells. **(E-F)** Tagitinin C induced G2/M cell cycle arrest in HCT116 cells. Representative images **(E)** and quantifications of populations of HCT116 cells in the cell cycle **(F)** after cells were treated with increasing concentration of tagitinin C (5, 10, 20 µM) for 24 h. Data were presented as Mean ± SD. Scale bar indicates 100 µm. Statistical analysis was carried out between tagitinin C-treated group and DMSO group: * *p* ≤ 0.05, ** *p* ≤ 0.01, *** *p* ≤ 0.001.

We next investigated the effect of tagitinin C on colony formation in HCT116 cells by clonogenic cell survival assay 28-30. As shown in Figure 2A-B, tagitinin C induced a significant concentration-dependent reduction in the number of colonies after 14 days treatment. The results indicated that tagitinin C significantly inhibited the clonogenic growth of HCT116 cells. Transwell migration assay was employed to investigate the effect of tagitinin C on cell migration ability of HCT116 cells (Figure 2C-D). The results suggested that tagitinin C significantly inhibited cell migration in a dose-dependent manner.

Further, we examined the effect of tagitinin C on cell cycle by flow cytometry. As shown in Figure 2E-F, significant changes in cell cycle were observed in HCT116 cells. As compared with control group, cells were arrested at G2/M phase in a dose-dependent manner. These data indicated that tagitinin C induced G2/M cell cycle arrest in HCT116 cells and might suppress the proliferation of cells by inducing cell cycle arrest.

### Tagitinin C induces ferroptosis in HCT116 cells

**Figure 3:**
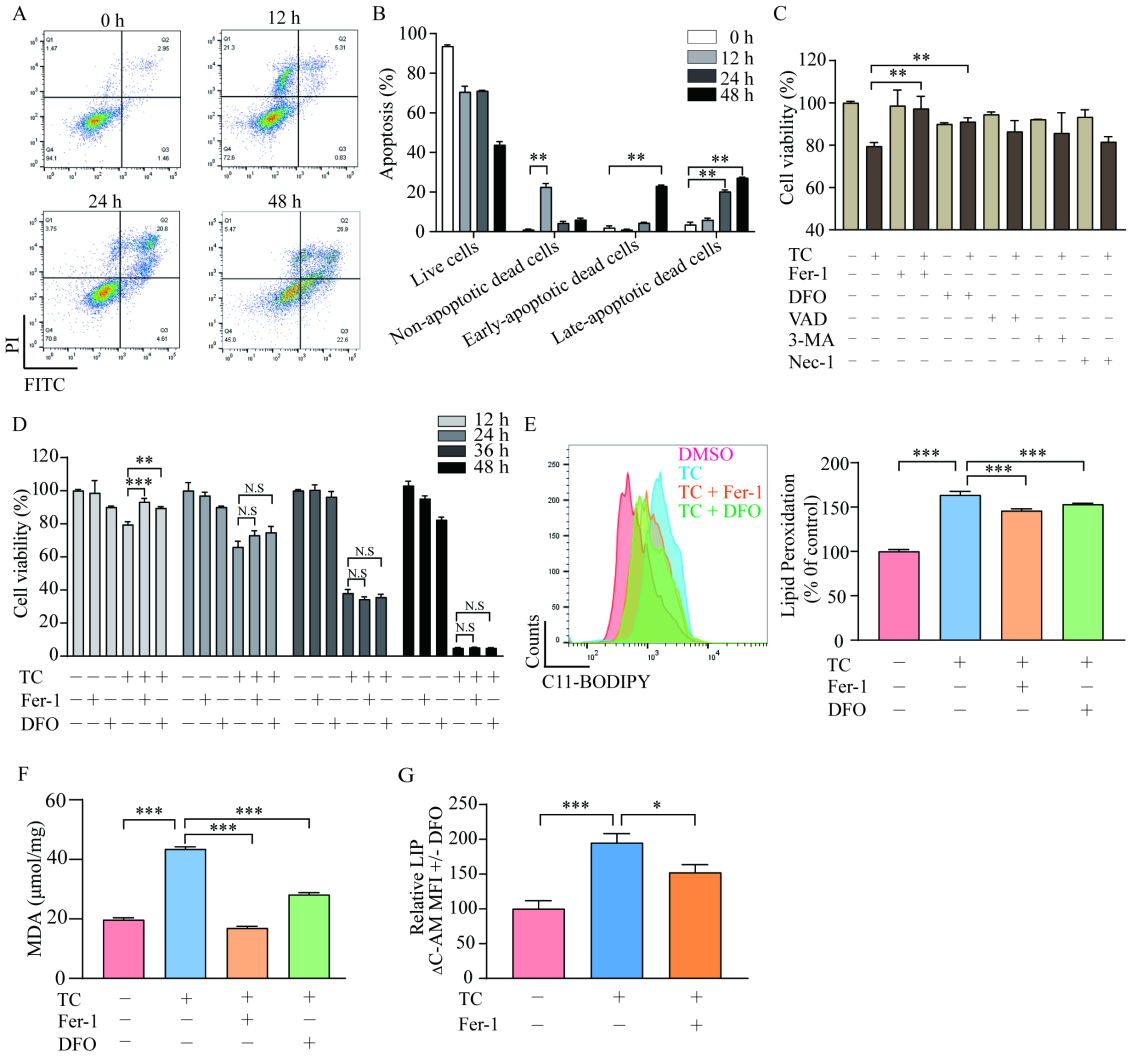
** Tagitinin C induces ferroptosis in HCT116 cells. (A-B)** Flow cytometric analysis of apoptosis in the HCT116 cells treated with tagitinin C (20 µM) after 0, 12, 24, 48 h. Image **(A)** and quantitative analysis for apoptotic cells **(B)** were presented. **(C)** Cell viability was measured using CCK-8 assay in control cells and cells treated with tagitinin C (20 µM) with or without Fer-1 (1 µM), DFO (5 µM), Z-VAD-FMK (20 µM), 3-MA (2 mM), and Nec-1 (50 µM) at 12 h. **(D)** Fer-1 (1 µM) and DFO (5 µM) rescued tagitinin C-induced cell death at 12 h. **(E)** Fer-1 and DFO blocked tagitinin C-induced lipid peroxidation, quantified using C11-BODIPY lipid probe using flow cytometry in HCT116 cells.** (F)** Quantification of cellular MDA levels using the TBA method. **(G)** Quantification of cellular LIP levels using the calcein-AM (C-AM) method. The mean fluorescence intensity (MFI) of C-AM is subtracted from the MFI of C-AM treated with DFO. Data were presented as Mean ± SD. Statistical analysis was carried out between tagitinin C-treated group and DMSO group: * *p* ≤ 0.05, ** *p* ≤ 0.01, *** p ≤ 0.001.

To investigate whether tagitinin C induces apoptosis as previously reported, the cell death was detected by flow cytometry analysis using Annexin V and PI staining (Figure 3A-B). Results showed that tagitinin C could induced apoptosis after 24 or 48 hours treatment. Interestingly, for short time treatment (12 hours) with tagitinin C in HCT116 cells, a portion of the cells gathered in the area of Annexin V^-^ / PI ^+^ (Figure 3A). This result suggested that tagitinin C may have induced or may induce a different type of cell death other than apoptosis within 12 hours. To further investigate what kind of cell death was induced by tagitinin C in this short time, different cell death inhibitors were tested together with tagitinin C. As shown in Figure 3C, tagitinin C-induced cell death was attenuated by the pharmacological ferroptosis inhibitors, Fer-1 (Ferrostatin-1) 31 and DFO (Deferoxamine) 32, but not by VAD (Z-VAD-FMK, apoptosis inhibitor), 3-MA (autophagy inhibitor) and Nec-1 (necrostatin-1, necroptosis inhibitor). This result indicated that tagitinin C-induced cell death within 12 hours is non-apoptotic and non-necroptotic cell death and might be the novel programmed cell death - ferroptosis. However, Fer-1 and DFO cannot attenuate the cell death induced by tagitinin C-induced cell death for longer than 24 h or longer time (Figure 3D), suggesting that tagitinin C-induced apoptosis might be dominant for longer time treatment. All these results indicated that tagitinin C-induced early cell death might be ferroptosis while apoptosis might be induced upon longer time treatment.

Lipid peroxidation, a key event of ferroptosis 33, was increased by tagitinin C at 12 h, which was reversed partly by Fer-1 and DFO (Figure 3E), indicating that the addition of tagitinin C enhanced lipid peroxidation. Similar results were observed with regard to the changes in the level of malondialdehyde (MDA) in HCT116 cells induced by tagitinin C (Figure 3F). MDA is the most prevalent byproduct of lipid peroxidation 34. It is known that intracellular iron contributes to the ferrous iron accumulation that leads to cell cytotoxicity via specific ROS generation 35, which is responsible for ferroptosis. Next, we assessed how tagitinin C regulates cellular iron in HCT116 cells. Both free and chelatable iron is the so-called labile iron pool (LIP) 36. LIP levels were upregulated by tagitinin C at 12 h but significantly less so in the presence of Fer-1 (Figure 3G). Further, we found that after the tagitinin C treatment within 12 hours, lipid peroxidation, MDA and LIP (Figure S1B-D) were up-regulated to various degrees. These results indicated that tagitinin C-induced HCT116 early cell death was accompanied with elevated lipid peroxidation, MDA and LIP, which are characteristic features of ferroptosis.

### Tagitinin C induces ROS generation, which contributes to mitochondrial dysfunction

**Figure 4:**
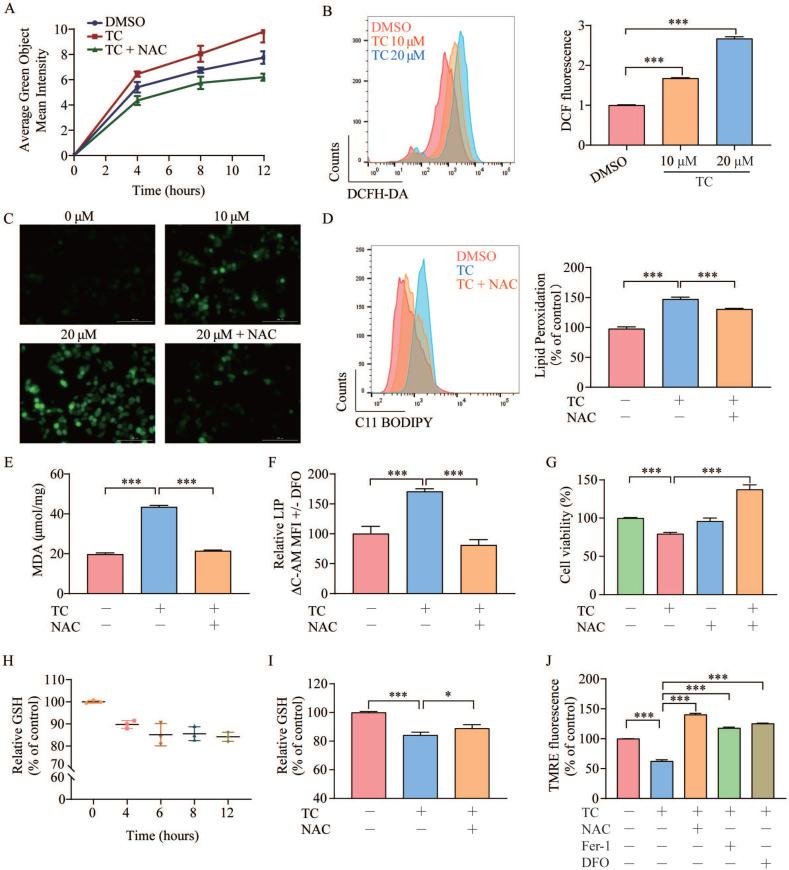
** Tagitinin C induces ROS generation and contributes to mitochondrial dysfunction. (A-C)** DCFH-DA probe was used to detected ROS levels in HCT116 cells by Incucyte S3 **(A)**, flow cytometry **(B)** and fluorescence microscope **(C)** (magnification, ×20) respectively. **(D-E)** Tagitinin C (20 µM) increased the level of lipid peroxidation **(D)**, MDA** (E)** and LIP **(F)**, which could be blocked by NAC (200 µM). **(G)** Cell viability was measured using CCK-8 assay in control cells and cells treated with tagitinin C with or without NAC (200 µM) at 12 h. **(H)** Quantification of the reduced cellular GSH levels using the MCB method. **(I)** Tagitinin C (20 µM) decreased the level of GSH, which could be blocked by NAC (200 µM). **(J)** Tagitinin C (20 µM) decreased the TMRE fluorescence in HCT116 cells, which could be blocked by NAC (200 µM), Fer-1 (1µM) and DFO (5 µM). Data were presented as Mean ± SD. Scale bar indicates 100 µm. Statistical analysis was carried out between tagitinin C-treated group and DMSO group: * *p* ≤ 0.05, ** *p* ≤ 0.01, *** *p* ≤ 0.001.

Ferroptosis depends on ROS production for its cytotoxicity 11, 37. To investigate the role of ROS in tagitinin C-induced ferroptosis, we first used the DCFH-DA probe to real-time monitor the total cellular ROS generation, and then used incucyte (Figure 4A) and flow cytometry (Figure 4B) to detect changes of fluorescence intensity after cells were treated with tagitinin C. The results revealed that tagitinin C rapidly triggered ROS production in HCT116 cells in a time- and concentration-dependent manner, this phenotype can be efficiently reversed by an antioxidant, N-acetyl-L-cysteine (NAC) 38. Fluorescence signals detected by microscopy were consistent with the above results (Figure 4C). NAC not only effectively inhibited the generation of ROS and subsequent lipid peroxidation, MDA and LIP (Figure 4D-F), but also rescued the cell death induced by tagitinin C in HCT116 cells at 12 h (Figure 4G).

GSH is an antioxidant that plays a crucial role in maintaining the redox balance and defending against oxidative stress (including ROS) in cells 39. We evaluated intracellular GSH levels in HCT116 cells after treatment with tagitinin C within 12 hours. The results showed that GSH level was decreased within 12 h (Figure 4H), and this decrease was also attenuated when cells were treated with the combination of tagitinin C and NAC (Figure 4I). In addition, ROS is a critical factor in maintaining mitochondrial homeostasis. Mitochondrial membrane potential (ΔΨm) is often used to measure mitochondrial function, and a loss of ΔΨm indicates mitochondrial dysfunction 40. TMRE is a dye widely used in the detection of ΔΨm. Fluorescence intensity measured by microplate reader showed that TMRE fluorescence in HCT116 cells discreased with tagitinin C treatment, and this decrease was also attenuated when cells were treated with the combination of tagitinin C and NAC, Fer-1 and DFO (Figure 4J). This result indicated that a loss of ΔψM was caused by tagitinin C and might result in dysfunction of mitochondria.

### Tagitinin C activates Nrf2-HO-1 signaling pathway

**Figure 5:**
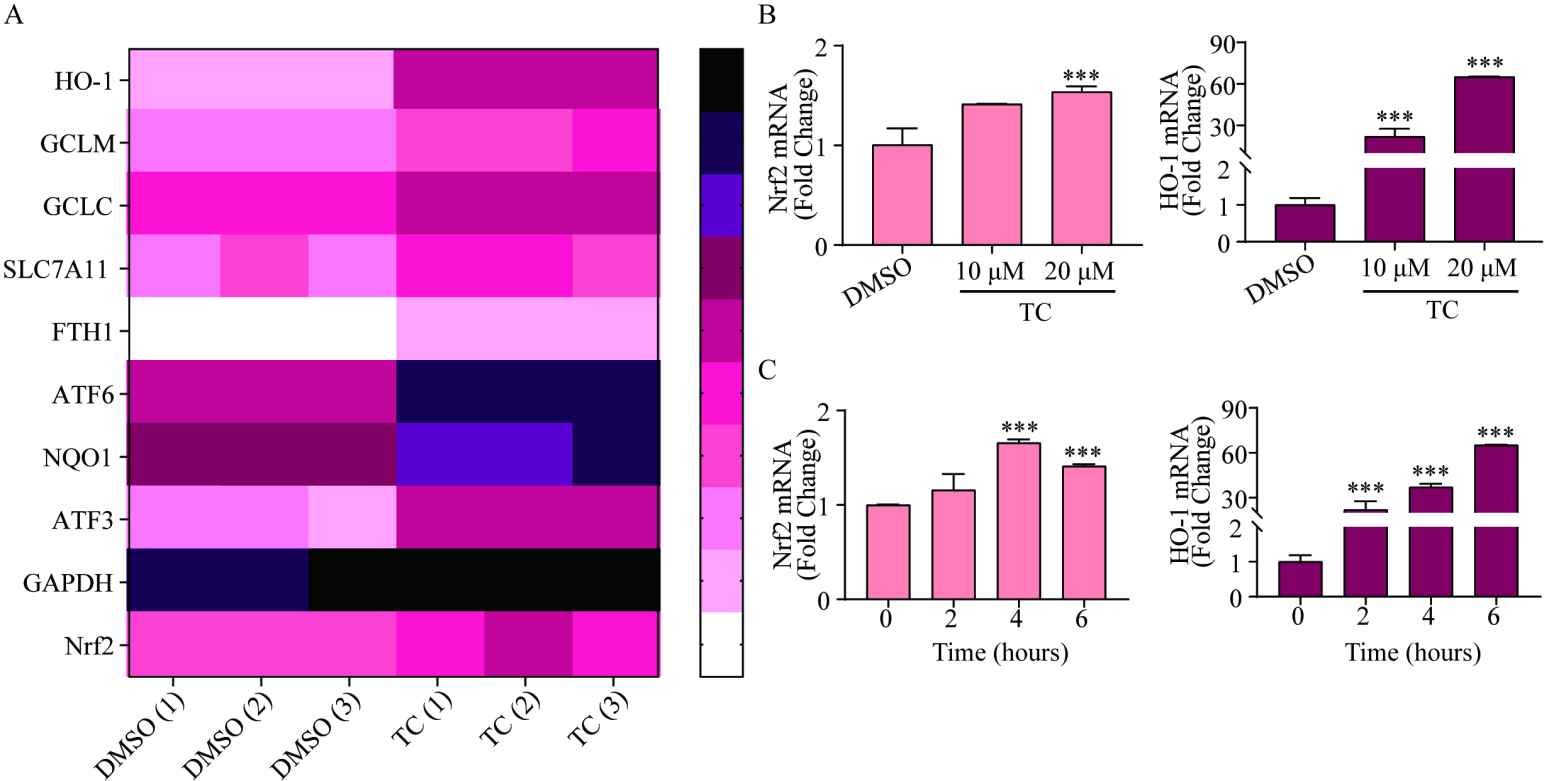
** Tagitinin C activates Nrf2-HO-1 signaling pathway. (A)** Heat map showed differentially up-regulated genes in response to tagitinin C treatment measured using RNA-seq analysis. Low expression is depicted in white, and high expression is depicted in black. **(B-C)** Nrf2 and HO-1 mRNA was measured at indicated concentrations or time after treatment of tagitinin C. Data were presented as Mean ± SD. Statistical analysis was carried out between tagitinin C-treated group and DMSO group: * *p* ≤ 0.05, ** *p* ≤ 0.01, *** p ≤ 0.001.

In order to investigate the underlying mechanism of tagitinin C-induced ferroptosis, RNA-sequencing analysis was employed. We found that tagitinin C-mediated oxidative stress involved the activation of the Nrf2 signaling pathway (Figure 5A). Nrf2, is a well-known upstream element of the genes that are up-regulated in response to ROS 41 and ER stress 42,43. Nrf2 is a transcription factor and phosphorylated Nrf2 translocates into the nucleus to transactivate its target genes, including HO-1, GCLM, GCLC, SLC7A11, FTH1, ATF6, NQO1, ATF3, and GAPDH, as well as Nrf2 itself. Our results showed that tagitinin C significantly increased expression of Nrf2 and HO-1 in HCT116 cells at the mRNA level in a concentration-dependent manner at 6 h after addition of tagitinin C (Figure 5B). Further, at the concentration of 20 μM tagitinin C, mRNA expression level of Nrf2 reached a peak at 4 h (Figure 5C), and mRNA expression level of HO-1 was also upregulated by tagitinin C in a time-dependent manner within 6 h (Figure 5C). At the same time, we tested the mRNA levels of Nrf2 and HO-1 in HCT116 cells for 24 h and 48 h after the treatment of tagitinin C, and the mRNA levels of Nrf2 and HO-1 decreased (Figure S1E-F). This result suggested that Nrf2-HO-1 signaling pathway was activated at early timepoints when treated with tagitinin C, which might play a critical role in inducing ferroptosis within 12 hours.

### Tagitinin C induces ER stress, which mediates Nrf2-HO-1 activation

**Figure 6:**
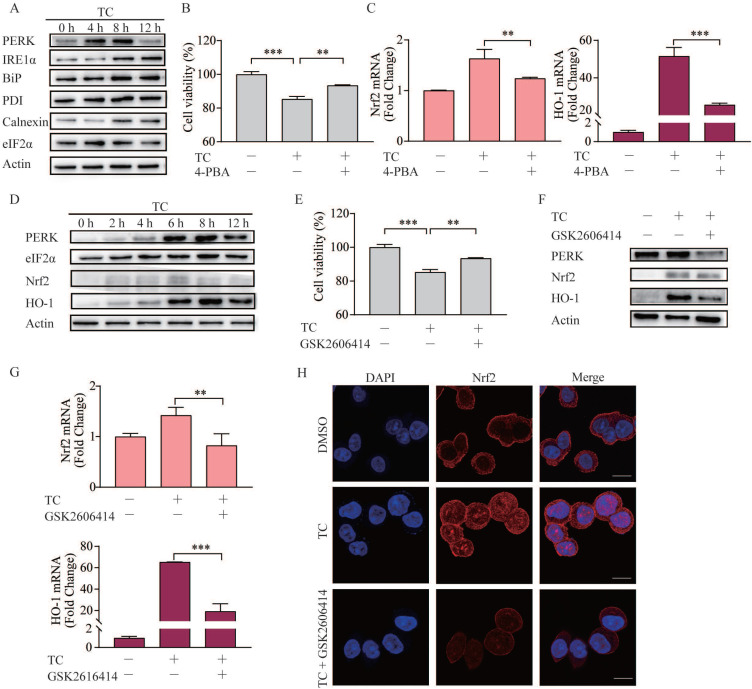
** Tagitinin C induces ER stress, which mediates Nrf2-HO-1 activation. (A)** HCT116 cells stimulated ER stress. HCT116 cells were treated with 20 µM tagitinin C for the indicated time. ER stress-related proteins were determination by Western blot analysis. **(B)** Cell viability was measured using CCK-8 assay in control cells and cells treated with tagitinin C (20 µM) with/without 4-PBA (20 µM). **(C)** HCT116 cells were treated with tagitinin C (20 µM) with 4-PBA (20 µM) for 6 h, the cells were collected and used for Nrf2 and HO-1 mRNA determination. **(D)** HCT116 cells were treated with 20 µM tagitinin C for the indicated time intervals. Cells were collected for PERK, eIF2a, Nrf2, and HO-1 determination by Western blot analysis. **(E)** Cell viability was measured using CCK-8 assay in control cells and cells treated with tagitinin C (20 µM) with/without GSK2606414 (5 µM). **(F)** HCT116 cells were treated with 20 µM tagitinin C and/or GSK2606414 for 8 h, the cells were collected and used for Western blot analysis. **(G)** HCT116 cells were treated with tagitinin C (20 µM) with GSK2606414 for 6 h, the cells were collected and used for Nrf2 and HO-1 mRNA determination. **(H)** HCT116 cells were grown on coverslips and treated with tagitinin C. Tagitinin C-induced Nrf2 nuclear translocation was observed under confocal microscope by IF. Cells were stained with Nrf2 (red) and DAPI (blue) (magnification, ×100). Data were presented as Mean ± SD. Scale bar indicates 100 µm. Statistical analysis was carried out between tagitinin C-treated group and DMSO group: * *p* ≤ 0.05, ** *p ≤* 0.01, *** *p* ≤ 0.001.

ER stress can be triggered by natural products and anticancer chemicals 44, including erastin 45, and ER stress plays important role in the cross talk between ferroptosis and other forms of cell death 46. Here, we showed that tagitinin C-induced ER stress in a time-dependent manner in HCT116 cells (Figure 6A). To investigate whether ER stress was involved in tagitinin C-induced ferroptosis, we used 4-PBA, an ER stress inhibitor, together with tagitinin C to check the cell viability and genes in Nrf2 signaling pathway. As shown in Figure 6B-C, 4-PBA effectively inhibited the tagitinin C-induced cell death and mRNA expression level of Nrf2 and HO-1.

Further, we investigated the key mediator of the tagitinin C-induced ER stress and Nrf2-HO-1 signaling pathway. In previous reports, Nrf2 can be directly phosphorylated by PERK 47. Our results from western blot assay showed that the abundance of PERK, Nrf2 and HO-1 increased within 12 h after tagitinin C treatment (Figure 6D). To investigate whether PERK mediated the activation of Nrf2-HO-1 signaling pathway by tagitinin C, we combined GSK2606414 (PERK inhibitor) with tagitinin C in the culture medium of HCT116 cells. The results showed that GSK2606414 decreased tagitinin C-induced cell death (Figure 6E), and also attenuated the upregulation of Nrf2 and HO-1 induced by tagitinin C at both protein and mRNA expression level (Figure 6F-G). As mentioned before, Nrf2 is a transcription factor and phosphorylated Nrf2 translocates into the nucleus to transactivate its target genes, including HO-1. As shown in Figure 6H, tagitinin C upregulated the expression of Nrf2 in cells and promoted the nuclear translocalization of Nrf2, whereas GSK2606414 significantly inhibited Nrf2 expression as detected by Immunofluorescence (IF). All these results indicated that PERK might be an important mediator of tagitinin C-induced activation of Nrf2-HO-1 signaling pathway.

### Combination of erastin and tagitinin C synergistically induces ferroptotic cell death

**Figure 7:**
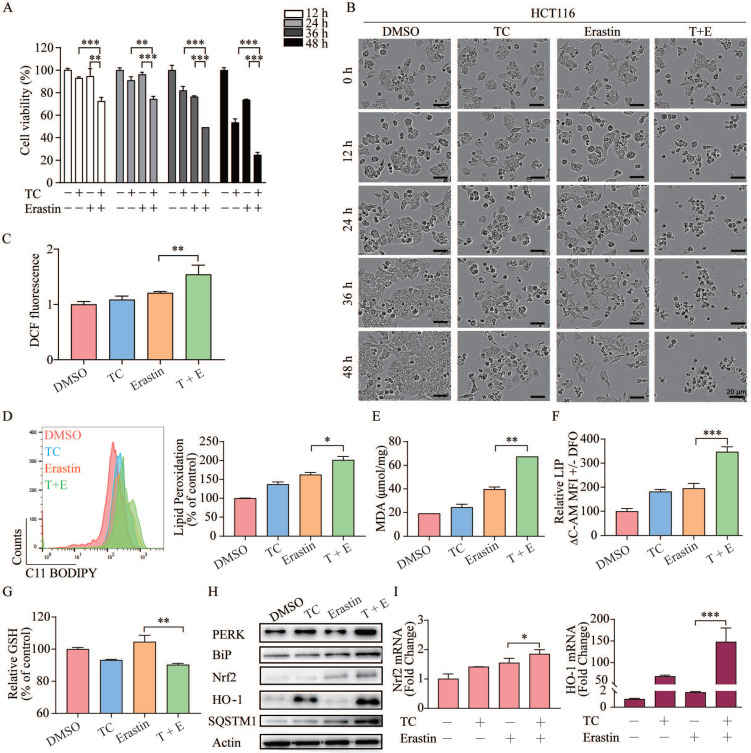
** Combination of erastin and tagitinin C synergistically induces cell death. (A)** Cell viability was measured using MTT assay in HCT116 cells treated with tagitinin C with or without erastin at 12, 24, 36 and 48 h. **(B)** The HCT116 cell morphology after treatment with concentration of tagitinin C (10 µM) and/or erastin (20 µM) at 0, 12, 24, 36, 48 h (magnification, ×10). **(C)** The contents of the cellular ROS. **(D-F)** The contents of the cellular lipid peroxidation **(D)**, MDA **(E)**, LIP **(F)** and GSH **(G)** in HCT116 cells at 12 h under tagitinin C (10 μM) and/or erastin (20 μM) were determined. **(H)** HCT116 cells were treated with tagitinin C (10 µM) and/or erastin (20 μM) for 12 h, the cells were collected and used for Western blot analysis. **(I)** HCT116 cells were treated with tagitinin C (10 µM) and/or erastin (20 µM) for 6 h, the cells were collected and mRNA level of Nrf2 and HO-1 were determination. Data were presented as Mean ± SD. Scale bar indicates 40 µm. Statistical analysis was carried out between tagitinin C-treated group and DMSO group: * *p* ≤ 0.05, ** *p* ≤ 0.01, *** *p* ≤ 0.001.

As erastin mainly functions through inhibition of cystine-glutamate reverse to elicit ferroptosis, whereas tagitinin C may functions through activation of Nrf2-HO-1 signaling pathway. There is possibility that combined treatment of erastin and tagitinin C could enhance tumoricidal efficacy as a novel therapeutic strategy. To investigate whether tagitinin C combined with erastin could have synergistic effect on cell death induction, we examined the viability of HCT116 cells and their morphological changes after addition of tagitinin C (10 µM) and erastin (20 µM) at various time points. The MTT assays revealed that HCT116 cells treated with both compounds exhibited greater growth inhibition (Figure 7A). Compusyn was used to calculate the combination index (CI) values of erastin/tagitinin C cotreatments in HCT116 cells, the CI value was 0.72662 when tagitinin C (10 µM) and erastin (20 µM) was used, which meant moderate synergism 48. When used alone, erastin had no significant inhibitory effect on the proliferation of HCT116 cells. However, when erastin was used together with tagitinin C, cells shrank significantly and lost normal cell morphology (Figure 7B). Similarly, at 12 h cells treated with erastin or erastin and tagitinin C showed increases in ROS generation of 21.16% and 54.36%, respectively (Figure 7C). Consistently, the level of lipid peroxidation, MDA and LIP further increased (Figure 7D-F), whereas the level GSH decreased when treated with both erastin and tagitinin C (Figure 7G). Given that erastin can also induce ER stress, we investigated whether there was a synergistic effect on ER stress induced by erastin in combination with tagitinin C. The results indicated that the protein expression levels of PERK and BiP were increased after being treated with the combination of erastin and tagitinin C (Figure 7H). Further, both protein expression levels and mRNA expression levels of Nrf2 and HO-1 were further up-regulated after tagitinin C and erastin combined treatment (Figure 7I-J). In addition, we found that the expression level of SQSTM1/P62 protein increased significantly under the combined action of tagitinin C and erastin (Figure 7H), which has been reported to activate Nrf2 by degrading Keap1 49. This may explain the possible mechanism of the augmented activation of Nrf2-HO-1 signaling pathway by the combined treatment of erastin and tagitinin C. In summary, our results suggested that tagitinin C and erastin can synergistically induce ER stress and activate Nrf2-HO-1 signaling pathway, which leads to cell death, including ferroptosis.

## Discussion

At present, natural plant extracts have been regarded as an important source of anti-tumor drugs due to, not only their diversity of chemical structure and biological activity, but also their reduced overall toxicity and side effects compared to chemosynthetic drugs 50. Uncovering these categories of natural plant extracts and their functional mechanisms has become one of the research hotspots 51. Here, we report that tagitinin C, a natural compound isolated from *Tithonia diversifolia*, exhibited anti-tumor activities in colorectal cancer HCT116 cells. Mechanistically, tagitinin C could induce ferroptosis via ER stress-mediated activation of PERK-Nrf2-HO-1 signaling pathway.

Ferroptosis is a form of cell death mainly induced by intracellular iron accumulation and lipid peroxidation. Excessive iron contributes to ferroptosis through the production of ROS by Fenton reaction; nicotinamide adenine dinucleotide phosphate (NADPH)-dependent lipid peroxidation and GSH depletion also play crucial roles in the induction of ferroptosis 52. Our experimental results demonstrated that the levels of cellular ROS, lipid peroxidation**,** MDA and the labile iron pool were upregulated, whereas the GSH levels were downregulated in HCT116 cells by treatment of tagitinin C. Meanwhile, we tested the expression levels of the three important regulators of ferroptosis, POR, FSP1 and GPX4 53-55, the results showed that within 12 hours of tagitinin C treatment in HCT116 cells, the mRNA and protein expression levels of POR were upregulated, while the mRNA expression levels of FSP1 and GPX4 did not change significantly (Figure S1G-H). POR is oxidoreductase which can catalyze the oxidation of phospholipid, which induce membrane damage during ferroptosis. These findings confirmed that tagitinin C-induced ferroptosis contributed to its capability to promote cell death. We evaluated the role of tagitinin C in a ferroptosis resistant colorectal cancer cell line HCT116 56. It has been reported that HCT116 exhibit resistance to ferroptosis may through p53-dependent and p53-independent ways 56-58. Whether tagitinin C induce ferroptosis through p53 signaling pathway needs further evaluation.

By RNA-seq analysis of tagitinin C treated cells, we identified HO-1 as a possible target of tagitinin C. HO-1 is an endoplasmic reticulum (ER)-anchored enzyme that metabolizes heme into pro-oxidant ferrous iron, carbon monoxide and anti-oxidant biliverdin. HO-1 shows cytoprotective effects against various stress-related conditions 59. However, increasing numbers of studies have shown that high expression of HO-1 is an important positive regulator of ferroptosis which makes it an important candidate to mediate detrimental effects such as ferroptosis induction 60. Betulaceae extract induced ferroptosis in human colon cancer cells via the triggering of HO-1 expression 61. Erastin-induced ferroptotic cell death could be accelerated by HO-1 in HT-1080 fibrosarcoma cells and BAY 11-7085-induced ferroptosis was also mediated by HO-1 in breast cancer cells 62,63. Doxorubicin-induced ferroptosis in cardiomyocytes was also mediated by heme degradation, a result of Nrf2-mediated upregulation of HO-1 64. Withaferin A (WA), a natural ferroptotic agent, also induce ferroptosis in neuroblastoma by promoting iron accumulation and ROS production via HO-1 high expression 65. All these findings showed that the ferroptotic cell death can be induced by HO-1 through regulation of the amount of cellular iron and ROS. Apparently, HO-1 plays dual role in iron and ROS homeostasis, as well as ferroptosis induction, including cytoprotective effects or detrimental effects. Similar with the ferroptosis inducers mentioned above, tagitinin C also induced high expression of HO-1 within 6 hours, which led to cellular iron accumulation and ferroptosis. Thus, tagitinin C also functions as a ferroptosis inducer mainly through activation of Nrf2-HO-1 signaling pathway.

Production of ROS has been linked to ER stress 66,67. The endoplasmic reticulum (ER) is a specialized organelle for regulation of protein synthesis, folding, secretion, or posttranslational modification. In response to microenvironmental stimuli, protein misfolding in the ER occurs and accumulates, a process known as ER stress 68. To cope with ER stress, cancer cells initiate an unfolded protein response (UPR) through three different signaling pathways including PERK pathway. PERK is phosphorylated and activated by BiP 69,70, which subsequently phosphorylated Nrf2, a transcription factor. The latter dissociates from Nrf2/Keap1 complexes in the cytoplasm and then translocates into the nucleus, where Nrf2 interacts with antioxidant response element (ARE) and transactivates various genes, including HO-1, GCLM, SLC7A11, GCLC, FTH1, ATF6, NQO1, GAPDH and ATF3, as well as Nrf2 itself 71. Both ER stress and UPR are documented to be involved with the development of cancer and play an important role in ferroptosis 72,73. In our study, tagitinin C could induce ER stress, which lead to activation of PERK signaling pathway. ER stress inhibitor or PERK inhibitor effectively inhibited tagitinin C-induced cell death and activation of Nrf2-HO-1 signaling pathway. Besides ferroptosis, tagitinin C also induced apoptosis at later time points. Lipid peroxidation is a hallmark of ferroptosis. An excess of ROS, which propagates lipid peroxidation chain, also attacks biomembranes and subsequently leads to different types of cell death 74. ER stress has been reported to play important roles in the crosstalk between ferroptosis and apoptosis 75. ER stress-mediated activation of PERK-eIF2α-ATF4-CHOP pathway has been reported to induce both apoptosis and ferroptosis. Here, whether tagitinin C-induced ferroptosis and apoptosis is through the ER stress-mediated PERK signaling pathway needs further investigation.

Combined treatment of tagitinin C together with a well-known ferroptosis inducer, erastin, showed synergistic effects on cytotoxicity, including ferroptosis. Mechanistically, the effect caused by tagitinin C in inducing ferroptosis could be further amplified by erastin since erastin could also stimulate ER stress. The tumoricidal efficacy was enhanced by the combined treatment of tagitinin C and erastin, rendering ferroptosis insensitive HCT116 cells susceptible to ferroptotic cell death. In addition, we found tagitinin C also had synergistic cytotoxicity with ferroptosis inducer RSL3 (Figure S1I). This might provide insight into novel therapeutic strategy to overcome drug resistance in cancer.

In summary, in this study, we found that tagitinin C could induce ferroptosis by inducing ROS and stimulating ER stress, which leads to activation of PERK-Nrf2-HO-1 signaling pathway in HCT116 cells.

## Supplementary Material

Supplementary figures.

## Figures and Tables

**Figure 8:**
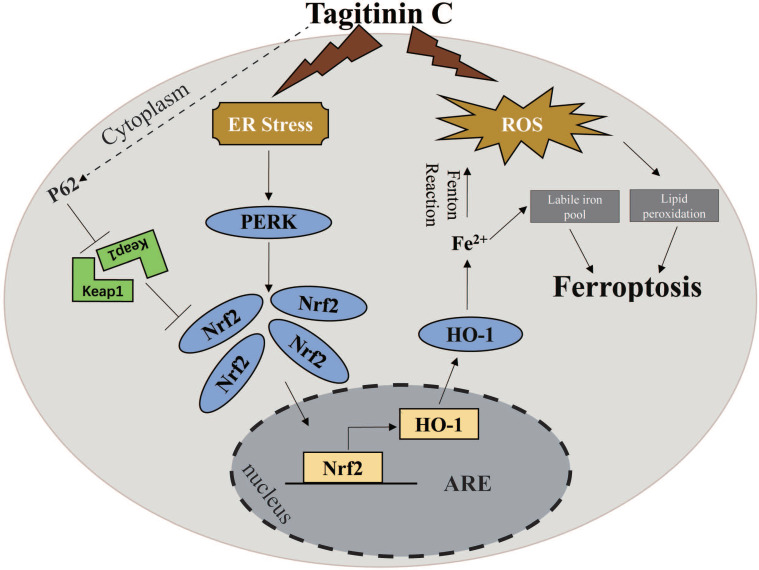
A proposed model showing that tagitinin C induces ferroptosis in HCT116 cells.
