# Supplementary material for: Tagitinin C induces ferroptosis through PERK-Nrf2-HO-1 signaling pathway in colorectal cancer cells
Source: Int J Biol Sci. 2021 Jun 26;17(11):2703–17. doi: 10.7150/ijbs.59404 (PMC8326123; doi:10.7150/ijbs.59404)

Tagitinin C induces ferroptosis through PERK-Nrf2-HO-1 signaling pathway in colorectal cancer cells.

Ruiran Wei [1], Yueqin Zhao [2], Juan Wang [2], Xu Yang [2], Shunlin Li [2], Yinyuan Wang [2], Xingzhi Yang [2], Jimin Fei [3], Xiaojiang Hao [2], Yuhan Zhao [2,*], Liming Gui [1,*] and Xiao Ding [2,*]

[1] Center for Tissue Engineering and Stem Cell Research, Guizhou Medical University 550004, Guiyang, China;

[2] State Key Laboratory of Phytochemistry and Plant Resource in West China, Kunming Institute of Botany, Chinese Academy of Sciences, 650201, Kunming, China

[3] Yunnan Cancer Hospital & The Third Affiliated Hospital of Kunming Medical University, 650118, Kunming, China

* Correspondence: zhaoyuhan@mail.kib.ac.cn (Y.Z.); guiliming@gmc.edu.cn(L.G.); dingxiao@mail.kib.ac.cn (X.D.)

Supplementary Figure S1

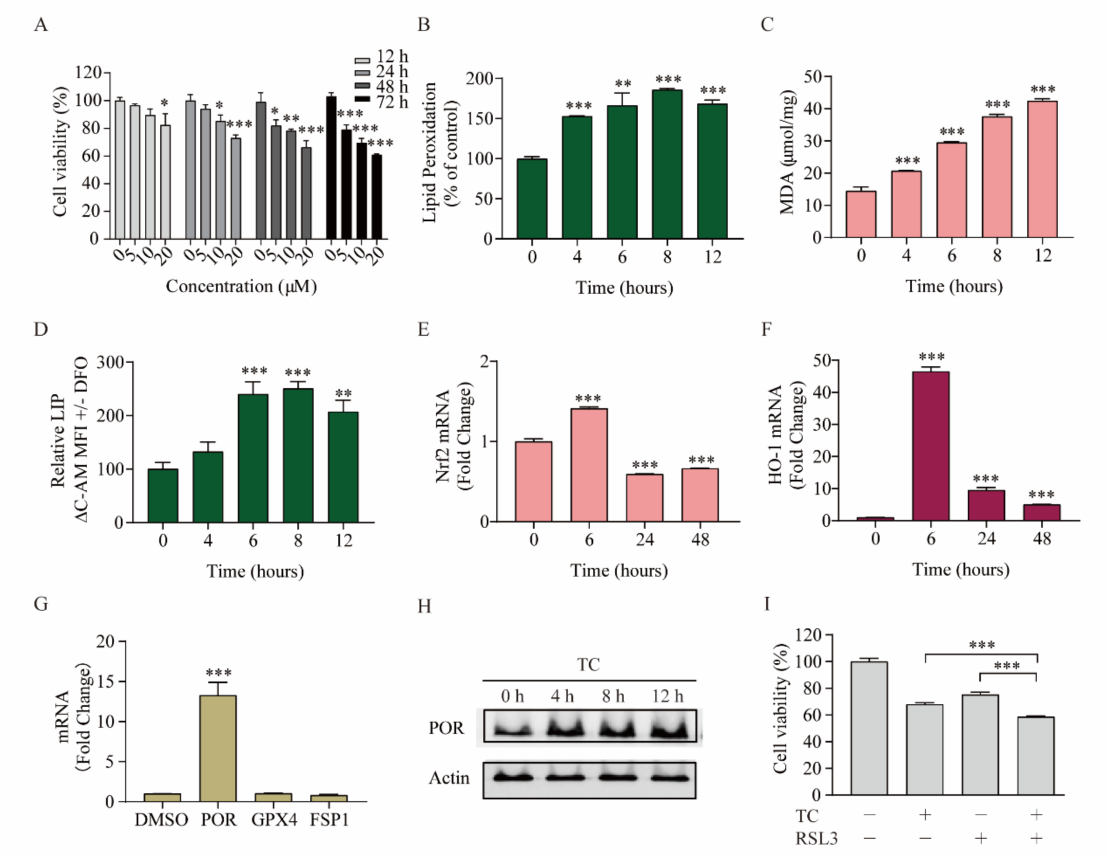

Figure S1. **(A)** Cell viability of HCT116 cells were measured by MTT assay after treatment with indicated concentration of erastin (0, 5, 10, 20 μM) at 12, 24, 48 and 72 h. **(B-D)** The contents of lipid peroxidation **(B)**, MDA **(C)** and the cellular LIP levels **(D)** in HCT116 cells at 0, 4, 6, 8 and 12 h under the concentration of 20 μM tagitinin C. **(E-F)** Nrf2 and HO-1 mRNA were measured at 24 and 48 h after treatment of tagitinin C (20 μM) in HCT116 cells. **(G)** POR, FSP1 and GPX4 mRNA were measured at 6 h after treatment of tagitinin C (20 μM) in HCT116 cells. **(H)** The ferroptosis-related

protein POR was determination by Western Blot. **(I)** Cell viability of HCT116 cells were measured by MTT assay after treatment with TC (10 μM) and RSL3 (20 μM) at 24 h.

Supplementary Figure S2

Figure 6A

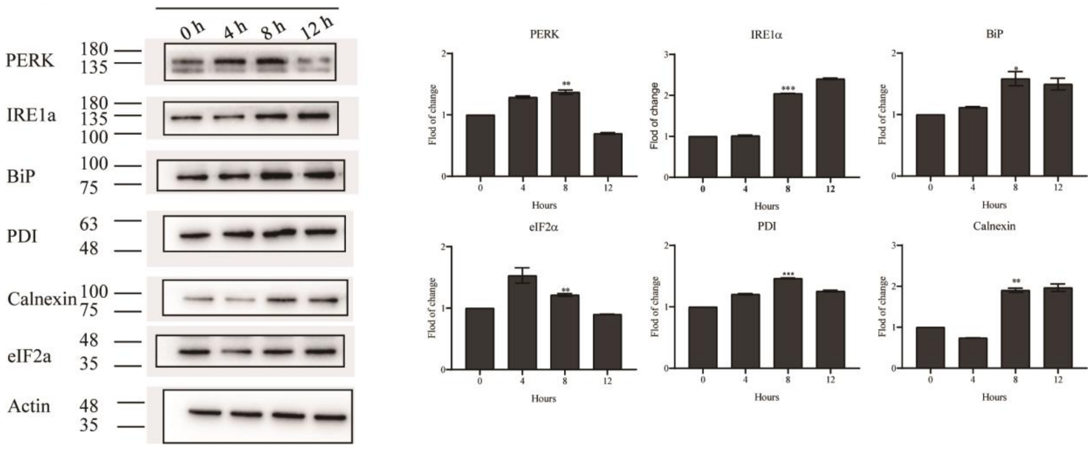

Figure 6D

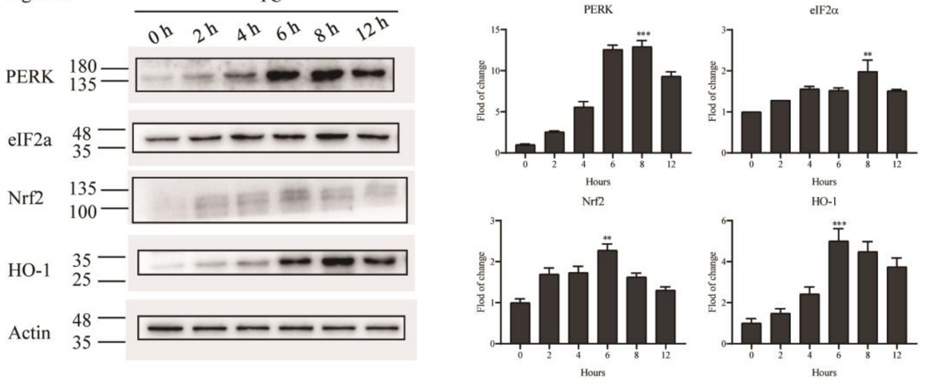

Figure 6F

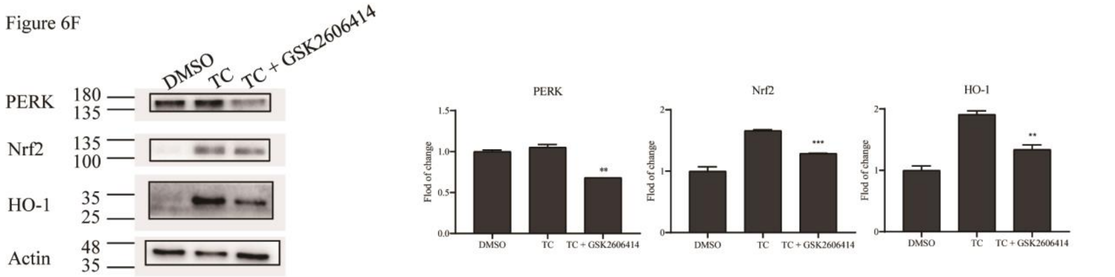

Figure 7G

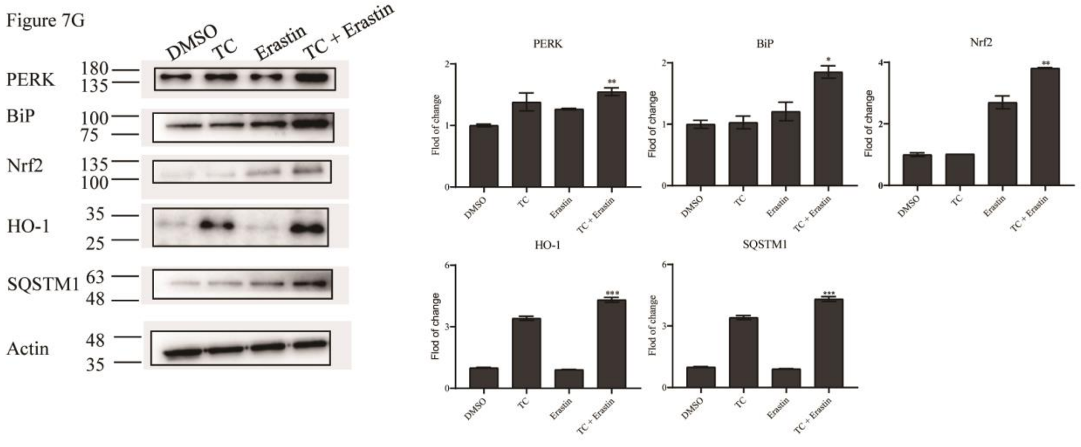

Supplement: Supplementary file 1 — Supplementary figures. [file ijbsv17p2703s1.pdf]
